# The Influence of Gastric Antral Ulcerations on the Expression of Galanin and GalR1, GalR2, GalR3 Receptors in the Pylorus with Regard to Gastric Intrinsic Innervation of the Pyloric Sphincter

**DOI:** 10.1371/journal.pone.0155658

**Published:** 2016-05-13

**Authors:** Michal Zalecki, Waldemar Sienkiewicz, Amelia Franke-Radowiecka, Magdalena Klimczuk, Jerzy Kaleczyc

**Affiliations:** Department of Animal Anatomy, Faculty of Veterinary Medicine, University of Warmia and Mazury in Olsztyn, Olsztyn, Poland; University of California, Los Angeles, UNITED STATES

## Abstract

Gastric antrum ulcerations are common disorders occurring in humans and animals. Such localization of ulcers disturbs the gastric emptying process, which is precisely controlled by the pylorus. Galanin (Gal) and its receptors are commonly accepted to participate in the regulation of inflammatory processes and neuronal plasticity. Their role in the regulation of gastrointestinal motility is also widely described. However, there is lack of data considering antral ulcerations in relation to changes in the expression of Gal and GalR1, GalR2, GalR3 receptors in the pyloric wall tissue and galaninergic intramural innervation of the pylorus. Two groups of pigs were used in the study: healthy gilts and gilts with experimentally induced antral ulcers. By double immunocytochemistry percentages of myenteric and submucosal neurons expressing Gal-immunoreactivity were determined in the pyloric wall tissue and in the population of gastric descending neurons supplying the pyloric sphincter (labelled by retrograde Fast Blue neuronal tracer). The percentage of Gal-immunoreactive neurons increased only in the myenteric plexus of the pyloric wall (from 16.14±2.06% in control to 25.5±2.07% in experimental animals), while no significant differences in other neuronal populations were observed between animals of both groups. Real-Time PCR revealed the increased expression of mRNA encoding Gal and GalR1 receptor in the pyloric wall tissue of the experimental animals, while the expression(s) of GalR2 and GalR3 were not significantly changed. The results obtained suggest the involvement of Gal, GalR1 and galaninergic pyloric myenteric neurons in the response of pyloric wall structures to antral ulcerations.

## Introduction

Galanin is a neuropeptide strongly involved in inflammation. Changes in the expression of galanin and its receptors are commonly observed in inflammatory diseases of different organs, including the gastrointestinal tract [1]. Neurons of the central and peripheral nervous system are known to express galanin and its levels are influenced by pathological processes occurring in the tissues supplied by these nerve cells. In the gastrointestinal tract galanin is expressed by myenteric and submucosal plexus neurons [2–5] and it is known to play a multiple role in the regulation of neurotransmission, mucosal secretion and smooth muscles contractions [6–11]. Galanin exerts its biological functions via the activation of three different galanin receptor subtypes—GalR1, GalR2, GalR3 [12]. Antral ulcerations are common disorders occurring in human and animals. Such ulcers are localized in the distal part of the stomach which is found close to the pyloric sphincter. Interestingly, gastric emptying is delayed only in patients with antral ulcerations, which is an unique occurrence [13] staying in contrast to the accelerated gastric emptying observed in patients with proximal gastric or duodenal ulcerations [14]. The pylorus, including its musculature called the pyloric sphincter, plays a key role in the regulation of gastric emptying. The pylorus is widely innervated by extrinsic and intrinsic nerves [15;16]. According to many authors, intrinsic (intramural) neurons that compose the enteric nervous system (ENS) are extremely important in the regulation of gastrointestinal motility [17;18]. Intramural gastric neuronal perikarya supplying the pylorus are localized in the myenteric and submucosal plexuses of the exact pylorus as well as in more proximal parts of the stomach. The latter ones contribute to so-called gastric descending nerve projections [19].

The gastric ulcerations are pathological processes accompanied by strong inflammatory reactions. Such pathological processes induce the specific response in the tissues. The response of neurons to pathological (or physiological) processes is widely defined as neuronal plasticity [20;21], which is manifested by changes in the expression of neuronal substances and receptors, among others galanin and its receptors [1;22;23]. These adaptive changes include both up and down regulation of transmitter expression and the induction of new genes in enteric nerve cells. They are developed not only to help enteric neurons to survive under pathological conditions but also to help the inflamed part of the gastrointestinal tract to recover. All these tissue responses are mainly observed in the place of injury or inflammation, however, the neighbouring tissues could be also affected. Interestingly, in the gastrointestinal tract the inflammatory process occurring in a certain site can influence the other, frequently even quite distant areas [24]. Antral ulcers, according to their specific localization, are known to influence the number and distribution of neurons contributing to gastric descending projections to the pyloric sphincter [25]. It seems to be reasonable to hypothesize that such ulcerations could additionally induce changes in the chemical coding of gastric intramural neurons (localized exactly in the pyloric wall and gastric descending projections) innervating the pylorus, and thus the number of such perikarya expressing galanin could be also changed. Moreover, the reaction of the pyloric wall to the released galanin could be additionally modified by changes in the expression of distinct galanin receptor’s subtypes. The confirmation of this assumption would clearly demonstrate the involvement of galanin and its receptors in the local gastric neuronal regulation of the pyloric function in subjects with antral ulcerations. It would also provide evidence that intrinsic gastric neurons and galanin may contribute to the gastric emptying disorders in individuals with such localization of gastric ulcers.

Pigs are animals of a great economic value, in which gastric ulcerations are common disorders resulting in a high mortality at the age of 3–6 months or slower growth of animals with severe gastric ulcerations [26–28]. Such consequences cause huge economic losses in many countries. Due to several morphological and physiological similarities to human organism, especially those regarding the gastrointestinal tract (swine is an omnivorous), the pig is considered to be the highly valuated animal model in biomedical research [29].

Data indicating the significant inhibitory effect of galanin and its receptors on gastrointestinal motility [9;30–32], gastric emptying [33] and sphincteric function [34] as well as those suggesting the considerable engagement of galanin and its receptors in inflammatory processes [1;23] seem to constitute good reasons to examine changes in the expression of galanin in the pyloric wall tissue and gastric intramural neurons supplying the pylorus in pigs with antral ulcerations.

Therefore, the aim of the present study was to verify changes in the expression of galanin in intramural gastric neurons supplying the pylorus (including the gastric descending neurons) in pigs with experimentally induced antral ulcerations as well as to quantitatively validate (by the Real-Time PCR) fluctuations in levels of mRNA encoding galanin and its receptors (GalR1, GalR2, GalR3) in the pyloric wall tissue in these animals.

## Materials and Methods

The handling of animals and all experimental procedures were in accordance with the rules of the National Ethics Commission for Animal Experimentation (Polish Ministry of Science and Higher Education). The protocol was approved by the Local Ethics Committee of the University of Warmia and Mazury in Olsztyn (Permit Number 76/2012) affiliated to the National Ethics Commission for Animal Experimentation (Polish Ministry of Science and Higher Education). All efforts were made to minimize animals suffering in each step of the experiment.

**Fig 1:**
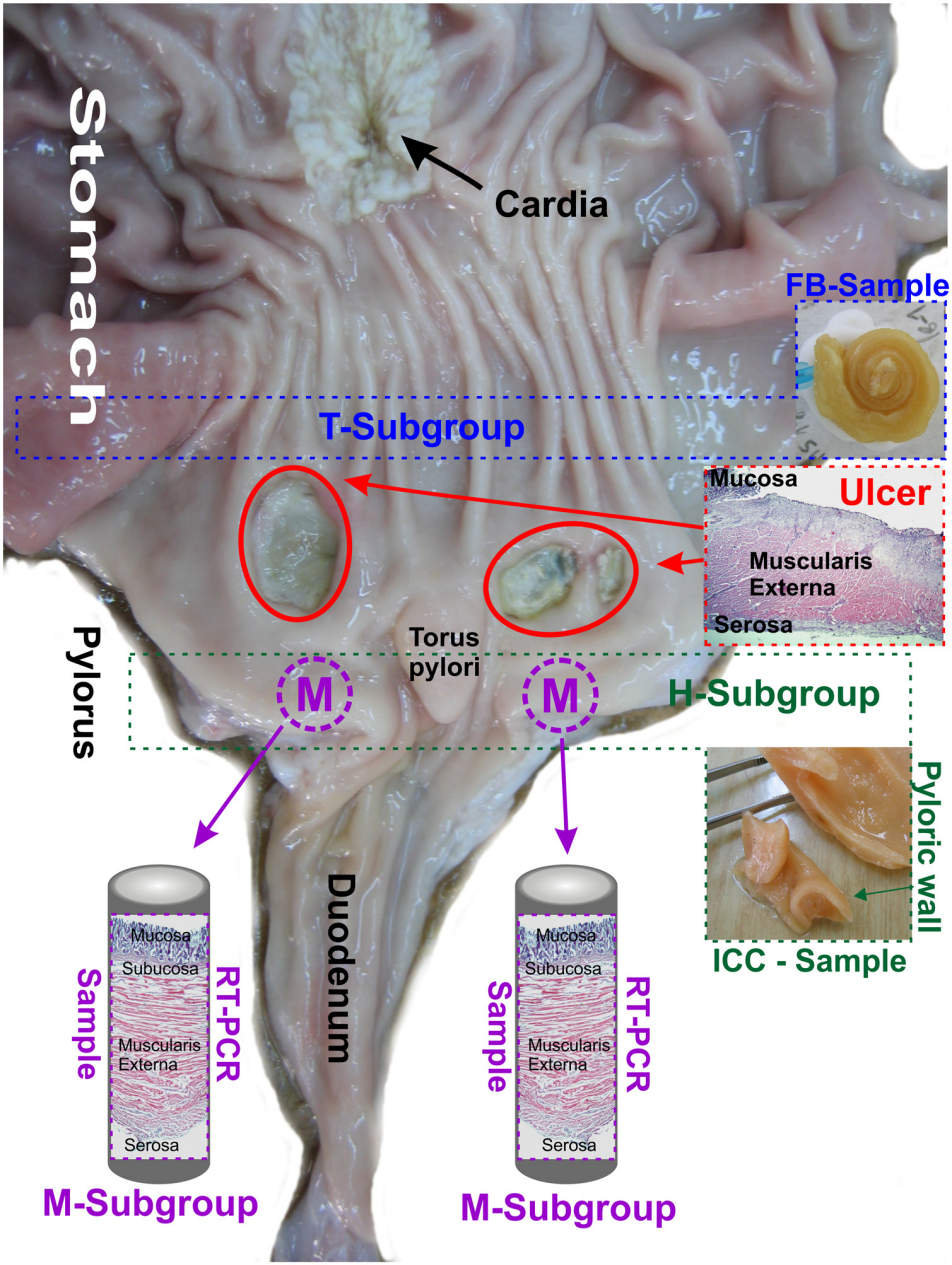
Tissue sampling. Diagram presenting the method of tissue sampling in the tracing (T), histochemical (H) and molecular (M) animal subgroups. The markings are applied in the picture presenting the interior surface of the "experimental animal" stomach which was cut along the greater curvature. Ulcers are indicated by red circles. Photomicrograph presenting the transverse section of the deeply penetrating stomach ulcer labelled with HE technique is shown in the red frame. Tissues containing Fast Blue (FB) traced perikarya were collected from the gastric antrum (blue frame) of the tracing subgroup pigs (T) and were cut into 20 μm thick cryostat consecutive microscopic sections. Tissues of the pyloric orifice wall (green frame) were collected from the histochemical subgroup of animals (H) and were cut into 20 μm thick cryostat microscopic sections. Tissues for Real-Time PCR were cut out bilaterally (about 0.5 cm from the torus pylori) from the pyloric orifice wall (violet circles with letter M) from the animals of the molecular subgroup (M). The circular-shaped samples, having a diameter of 1 cm, were cut transversally to the stomach wall by the use of a round cutter and comprised all layers of the pyloric orifice wall (violet frame).

The study was performed on sexually immature gilts of the Polish Large White breed (body weight approx. 20 kg) obtained from a commercial fattening farm (14–260 Lubawa, Poland). Since the present experiment is a part of the wider study, the tissues containing gastric descending neurons supplying the pyloric sphincter and retrogradely traced with Fast Blue (FB) tracer were collected during investigations described in the previous article [25]. The tissues of these animals were signed as tracing subgroup (T, n = 11, Fig 1—blue frame) and samples were collected from the stomach antrum of the control (n = 6) and experimental (n = 5) animals (detailed description of the methodology enclosed in the earlier article [25]).

The animals used in the current part of the study (n = 24) were divided into control (C, n = 12) and experimental (E, n = 12) groups. The application of two different types of research techniques (immunocytochemistry and Real-Time PCR) required two independent subgroups of animals, tissues of which were differently fixed. As a result, the animals (n = 24) were initially divided into histochemical (H, n = 12) and molecular (M, n = 12) subgroups. Each of these subgroups contained control (n = 6) and experimental (n = 6) pigs.

The pigs of the experimental group (n = 12) were pre-treated with azaperone (Stresnil, Janssen Pharmaceutica, Belgium, 0.4 mg/kg b.w., i.m.) and atropine (Polfa, Poland, 0.04 mg/kg b.w., s.c.) thirty minutes before the main anaesthetic was given. Then, the animals were generally anaesthetized with xylazine (Vetaxyl, Vet-Agro, Poland, 0.3 mg/kg b.w., i.m.) and ketamine (Bioketan, Vetoquinol Biowet, 15 mg/kg b.w., i.v., qs). Via the midline laparotomy the stomach was exposed and bilateral injections of 1 cm^3^ of 40% acetic acid solution were performed into the submucosal layer of the stomach antrum (as described in detail previously [25]) to evoke gastric ulcers (according to the acetic acid ulcer model procedure [35]). Finally, the midline abdomen incision wound was sutured, and secured by antibiotic (chlortetracycline, Animedazon^®^ Spray, aniMedica Gmbh, Germany) and micronized aluminum (Alu Spray, Arendonk, Belgium) spraying. The antibiotic (Betamox L.A., ScanVet, Poland, 15 mg/kg b.w., i.m.) and meloxicam (Metacam, Boehringer Ingelheim Vetmedica GmbH, Germany, 0.4 mg/kg b.w., i.m.) injections were performed to minimize the pain and suffering. The animals were moved to individual pens with unlimited access to water and kept for a week. On the next day after the surgery, the pigs had unlimited access to feed. The animals of the control group (n = 12) were kept for a week in the parallel pens and feed following the same feeding procedure. Finally all the pigs were deeply anaesthetized (as described previously) with the overdose of anaesthetic and sacrificed. The animals of the histochemical (H, n = 12) subgroup were transcardially perfused with a 4% solution of paraformaldehyde in 0.1M phosphate buffer (pH 7.4). Subsequent to perfusions, the stomachs were removed and cut along the greater curvature. All the gastric parts were thoroughly washed in PBS to remove food debris located on the mucosa. Then, 1 cm thick transverse section sample was taken from the pyloric orifice (Fig 1, green frame) and post–fixed in the same fixative as used for the perfusion (60 min.), rinsed in PBS for 2 days and transferred to and stored in 18% buffered (pH 7.4) sucrose solution for 3 weeks. Finally, 20 μm thick transverse cryostat consecutive microscopic sections were cut and mounted on chrome alum–gelatine–coated slides, air–dried and stored desiccated at -23°C until further processing.

The pigs of the molecular subgroup (M, n = 12) were deeply anaesthetized (as described previously) with the overdose of anaesthetic and exsanguinated. The stomach was dissected out, cut along the greater curvature. All the gastric parts were thoroughly washed in PBS. Then, two circular-shaped samples, having a diameter of 1 cm, were cut-out by use of a round cutter from all layers of the pyloric orifice wall (Fig 1, violet frame). The samples were taken from bilateral sidewall, about 0.5 cm from the torus pylori (Fig 1, violet circles with “M”). Afterwards, they were immersed in 4°C RNAlater^®^ (Ambion, USA) overnight and finally stored at -80°C until processing.

### Immunocytochemistry

Immunocytochemical stainings were performed on the pyloric wall (Fig 1—green frame) tissue slides collected from the pigs of subgroup H (n = 12), and on selected stomach antrum (Fig 1—blue frame) tissue slides collected from the animals of the subgroup T (n = 11) [which contained Fast Blue traced gastric neurons supplying the pyloric sphincter]. In order to provide certainty that none of the immunostained cell was counted twice, the slides processed were separated from each other by a minimum distance of 80 μm (greater than dimensions of the largest intramural perikarya). Next, the sections were double immunostained with a mixture of primary antibodies against pan-neuronal marker PGP 9.5 (mouse anti-PGP 9.5, dilution 1:600, code 7863–2004, clone 31A3, AbD Serotec) and galanin (rabbit anti-galanin, dilution 1:2500, code T4330, Peninsula Laboratories) and corresponding secondary antibodies (AlexaFluor 488, goat anti-mouse, dilution 1:500, code A11001 and AlexaFluor 555, goat anti-rabbit, dilution 1:500, code A-21428, Invitrogen, USA). The primary antibodies used in the study were recommended for application in the porcine tissues. All staining procedures and controls were performed according to the previously described protocol [36].

Tissue slides of the pyloric wall (Fig 1—green frame) collected from the pigs of the subgroup H were analysed under a fluorescent microscope equipped with a filter set for AlexaFluor 488/AlexaFluor 555. To determine the percentages of the myenteric and submucosal galanin-immunoreactive perikarya, at least 400 of PGP 9.5-positive cell bodies from each neuronal group in each animal stomach were analysed. The results were presented as average percentages ± SEM. The differences in the number of galanin-immunoreactive cells between the control and experimental animals as well as submucosal and myenteric plexuses were statistically analysed by the Student t-test, and considered to be significant at P < 0.05.

The dimensions of myenteric and submucosal Gal-positive perikarya were determined by confocal laser microscopy software measurements (Zen 2009, ver. 5.5.0.282, Zeiss) at a group of 50 PGP 9.5/Gal-immunoreactive neuronal somata (with a visible nucleus). The dimensions were measured perpendicularly and longitudinally to the longest axis of each perikaryon analyzed using fluorescence channel for PGP 9.5 staining (green). The results were presented as the average dimensions ± SEM.

Stomach antrum (Fig 1—blue frame) tissue slides collected from the pigs of the subgroup T were analysed under a fluorescent microscope equipped with a filter set for Fast Blue (to recognize the traced perikarya) and AlexaFluor 488/AlexaFluor 555 (to verify the expression of PGP 9.5 and galanin in the FB-positive neurons). To determine the percentages of galanin-immunoreactive traced perikarya at least 150 FB-positive neuronal somata from each animal stomach were analysed. The results were presented as average percentages ± SEM. The differences in the number of galanin-immunoreactive traced cells between the control and experimental pigs were statistically analysed by the Student t-test, and considered to be significant at P < 0.05. All statistical analyses were performed using GraphPad Software Inc., USA, ver. 6.

Finally, the selected slides were photographed with a confocal laser microscope (LSM700, Zeiss).

The Haematoxylin and Eosin stainings of the transverse sections of the pyloric wall tissue and ulcer tissue were carried out according to Ehrlich (Fluka; code 03972), and the slides were photographed by means of a stereo microscope (SteREO Discovery V8, Zeiss) equipped with a camera.

### Real-Time PCR

In order to obtain representative tissue samples from each animal stomach, 300 μg of every collected circular-shaped pyloric section, taken perpendicularly from all pyloric wall layers (Fig 1—violet circles and frames), was homogenized with 600 μl of fenozolone. Then, the appropriate volume of the liquid homogenate containing 50 μg of the tissue sample was used to isolate total RNA with a Total RNA Mini Plus kit (A&A Biotechnology, Poland). This has provided certainty that total RNA was isolated from all the pyloric wall layers in each sample of all the animals and that the samples were unified.

Reverse transcription was performed with 1.5 μg of total RNA and Maxima First Strand cDNA Synthesis Kit for RT-qPCR (code K1672, Thermo Fisher Scientific). Then, from each cDNA sample Real-Time PCRs were performed for the following genes: Gal, GalR1, GalR2, GalR3 and porcine glyceraldehyde 3-phosphate dehydrogenase (GAPDH) as the housekeeping gene, each in triplicate. The primers were designed with Primer-BLAST software (http://ncbi.nlm.nih.gov) and their detailed descriptions are enclosed in our previous study [37]. Sequences of primers are shown in Table 1. Composition of PCR mix was as follows: 10 μl of SYBR^®^ Select Master Mix (Thermo Fisher Scientific), 8 μl of ultra-pure DNase/RNase-free distilled water, 1 μl of cDNA preparation and 1 μl of 5 mM primer mix (reverse and forward, Sigma, USA). The PCR reaction was performed in 7500 fast Real-Time PCR system (Applied Biosystems, USA) with the thermal profile consisting of: initial denaturation 10 min at 95°C, denaturation 15 s at 95°C, and annealing 1 min at 60°C for 40 cycles. The data for galanin and its receptors expression were normalised against GAPDH using software 7500 v. 2.0.2 (Applied Biosystems, USA). Finally, data for each targeting gene were statistically compared between control and experimental animals with GraphPad Software Inc., USA, ver. 6 using the Student t-test, and were considered to be significant at P < 0.05.

**Table 1:** Sequences of primers used in Real-Time PCR.

Gene	Sequences of primers	Start position	Stop position	Product length	Sequence of origin (in Gene Bank)
GAPDH	Forward: TTCCACCCACGGCAAGTT	244	261	70	NM_001206359.1
GAPDH	Reverse: GGCCTTTCCATTGATGACAAG	293	313	70	NM_001206359.1
Gal	Forward: TGGGCCACATGCCATCGACA	356	375	94	NM_214234.1
Gal	Reverse: CGGCCTGGCTTCGTCTTCGG	430	449	94	NM_214234.1
GalR1	Forward: AGGATCACGGCGCACTGCCT	853	872	127	XM_003480426.2
GalR1	Reverse: GGGATTCCTTGCCAATGTGGCACT	956	979	127	XM_003480426.2
GalR2	Forward: GCCAAGCGCAAGGTAACGCG	688	707	126	XM_003484313.1
GalR2	Reverse: GTAGGTGGCGCGGGTAAGCG	794	813	126	XM_003484313.1
GalR3	Forward: GCACCACGCGCTCATCCTCT	750	769	122	XM_003355348.2
GalR3	Reverse: AGACCAGCGGGTTGAGGCAG	852	871	122	XM_003355348.2

The primers were designed using sequences of origin available in Gen Bank and Primer-BLAST software (http://ncbi.nlm.nih.gov).

## Results

Post-mortem examination of the stomach tissues collected from the experimental pigs revealed the presence of bilateral ulcers localized in the gastric antrum about 0.5–1.0 cm from the pyloric orifice (Fig 1 –red circles). The ulcers were of about 1.0–2.1 cm in a diameter and deeply penetrated into the muscular layer of the stomach wall (Fig 1—red frame).

**Fig 2:**
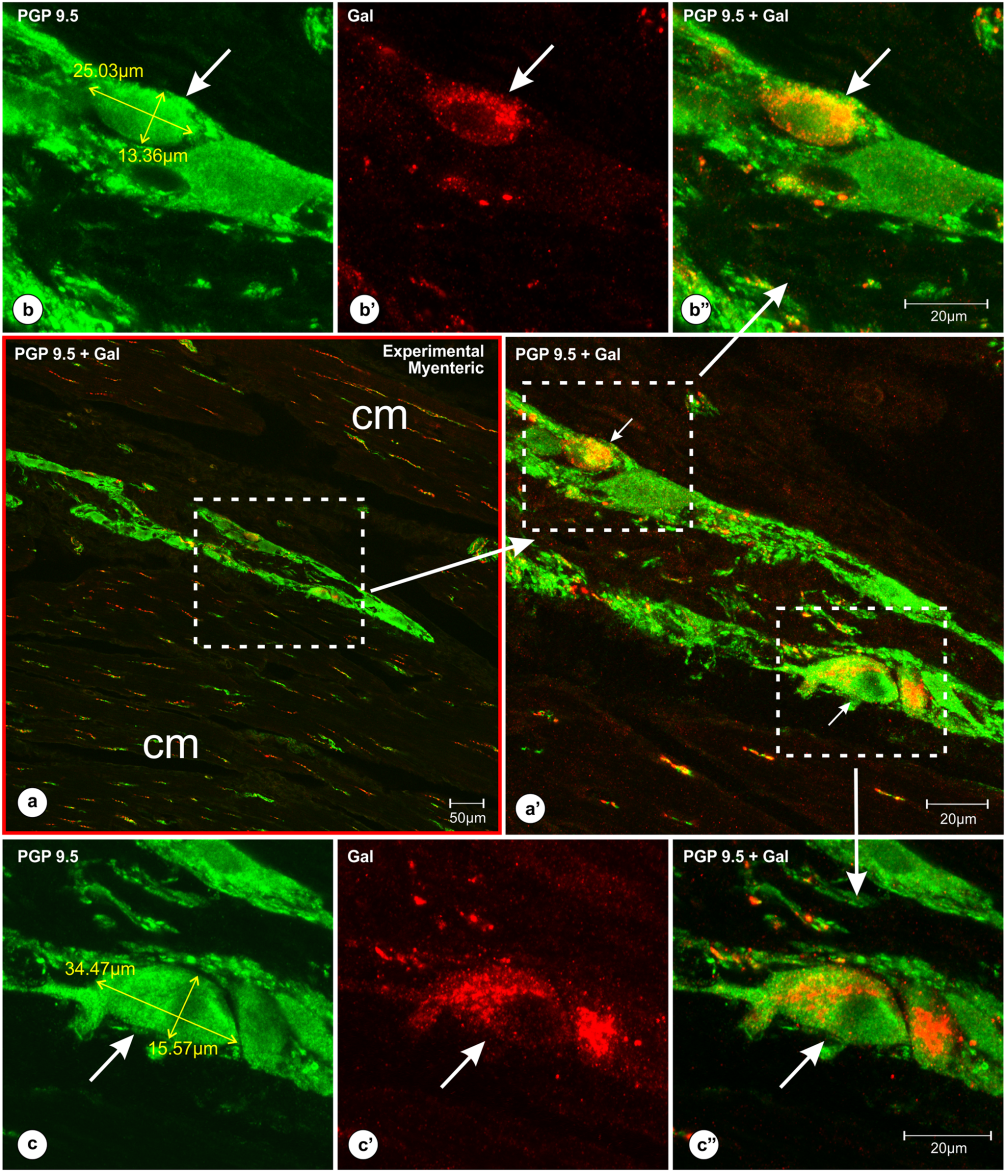
Localization of the myenteric plexus ganglia (containing Gal-immunoreactive perikarya) within the deep layers of the pyloric circular muscles. Set of microphotographs at different magnifications of the pyloric wall cross-section comprising myenteric plexus ganglion with PGP 9.5/Gal-immunoreactive neurons. The section was taken from the experimental pig of the subgroup H and double-immunolabeled with antibodies against PGP 9.5 (b, c) and galanin (b’, c’) [pictures (a, a’, b”, c”) present overlap of both fluorescence channels]. Low magnification picture (a, red frame) shows a myenteric plexus ganglion localized deep within the pyloric circular muscle layers [cm]. Medium magnification picture (a’) of the selected area [dotted line boarder from the picture (a)] presents Gal-immunofluorescent perikarya (arrows). High magnification pictures (b, b’, b’, c, c’, c”) show medium (b, b’, b”) and large (c, c’, c”) in a diameter PGP9.5/Gal-immunoreactive perikarya (arrows). The characteristic pattern of Gal-immunoreactivity observed in the neurons (b’, c’) blurred the outlines of the perikarya, precluding accurate measurements of the cell bodies using red channel [Gal immunostaining]. Thus, all the measurements of Gal-immunoreactive nerve cells were performed using the green channel [PGP 9.5 staining (b, c)]. Scale bars are included in the pictures.

The analysis of the pyloric wall (Fig 1—green frame) sections taken from the pigs of subgroup H and double immunostained with antibodies against PGP 9.5 and galanin revealed that most of the myenteric plexus ganglia containing Gal-immunoreactive perikarya were localized deep at different levels of the circular muscle layer (Fig 2a and 2a’). The pattern of galanin immunofluorescence visualized under high microscopic magnifications was very characteristic (Fig 2b’, 2b”, 2c’ and 2c”), Although the intensity of the galanin staining was differentiated, it was impossible to categorize the neurons in this respect because besides the very intensely or very weakly stained nerve cells many neuronal somata represented the diverse transitional forms.

**Fig 3:**
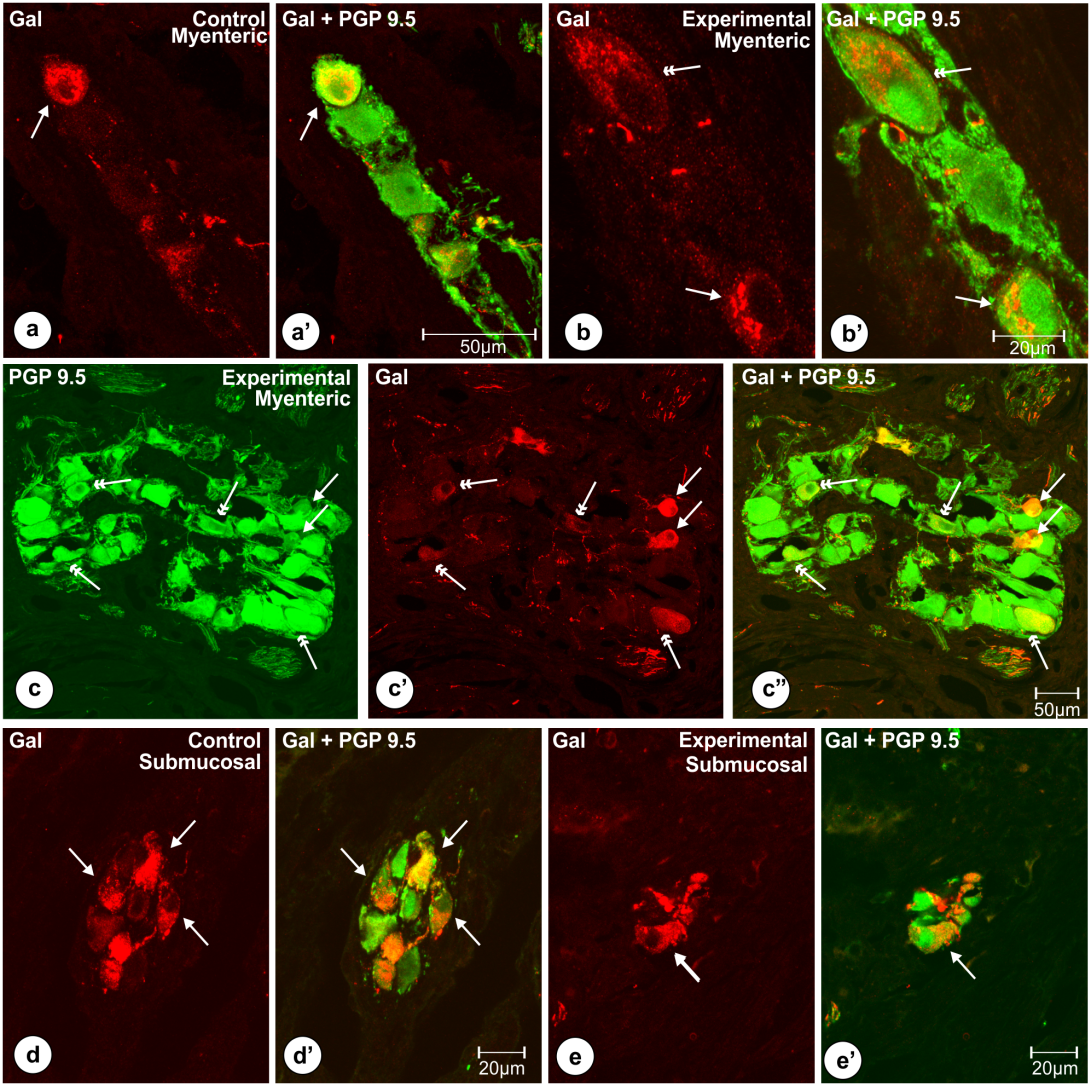
Typical characteristics (shapes, immunofluorescence) of Gal-immunoreactive perikarya. Set of photomicrographs showing shapes and patterns of immunofluorescence observed in the typical Gal-positive (single arrows) myenteric (a, a’, b’ b’, c, c’, c”) and submucosal (d, d’, e, e’) perikarya of the pyloric orifice wall in the control (a, a’, d, d’) and experimental (b, b’, c, c’, c”, e, e’) pigs of the subgroup H. In both groups of the animals most of the myenteric neurocytes immunoreactive to galanin (arrows) were round (a, c’) or oval (b, c’) and expressed medium to strong immunoreactivity. In the experimental animals (b, b’, c, c’, c”) some of Gal-positive perikarya (double arrows) seemed to express weak immunofluorescence and/or were larger in a diameter. Most of the submucosal neurocytes immunoreactive to Gal (arrows) in the control (d, d’) and experimental (e, e’) animals were oval and measured about 19.45 ± 0.65 x 11.33 ± 0.38 μm. Scale bars are included in the pictures.

The dimensions, shapes and immunoreactivity patterns of Gal-positive perikarya in corresponding groups of neurons in control and experimental animals were mostly similar. Most of the Gal-positive cells in the myenteric plexus ganglia (Figs 2b, 2b’, 2b”, 3a, 3a’, 3b, 3b’, 3c, 3c’ and 3c”) were round or oval in shape and measured 26.9 ± 1.06 x 17.12 ± 0.66 μm in a diameter, while submucosal neurons (Fig 3d, 3d’, 3e and 3e’) were mostly oval and had about 19.45 ± 0.65 x 11.33 ± 0.38 μm in a diameter. Most of the Gal-immunoreactive cells exhibited medium to strong immunofluorescence in both groups of the pigs. However, some cells in the myenteric plexus of the experimental animals seemed to exhibit less intense galanin immunofluorescence and/or were larger in a diameter (Figs 2c, 2c’, 2c”, 3b, 3b’, 3c, 3c’ and 3c”).

**Fig 4:**
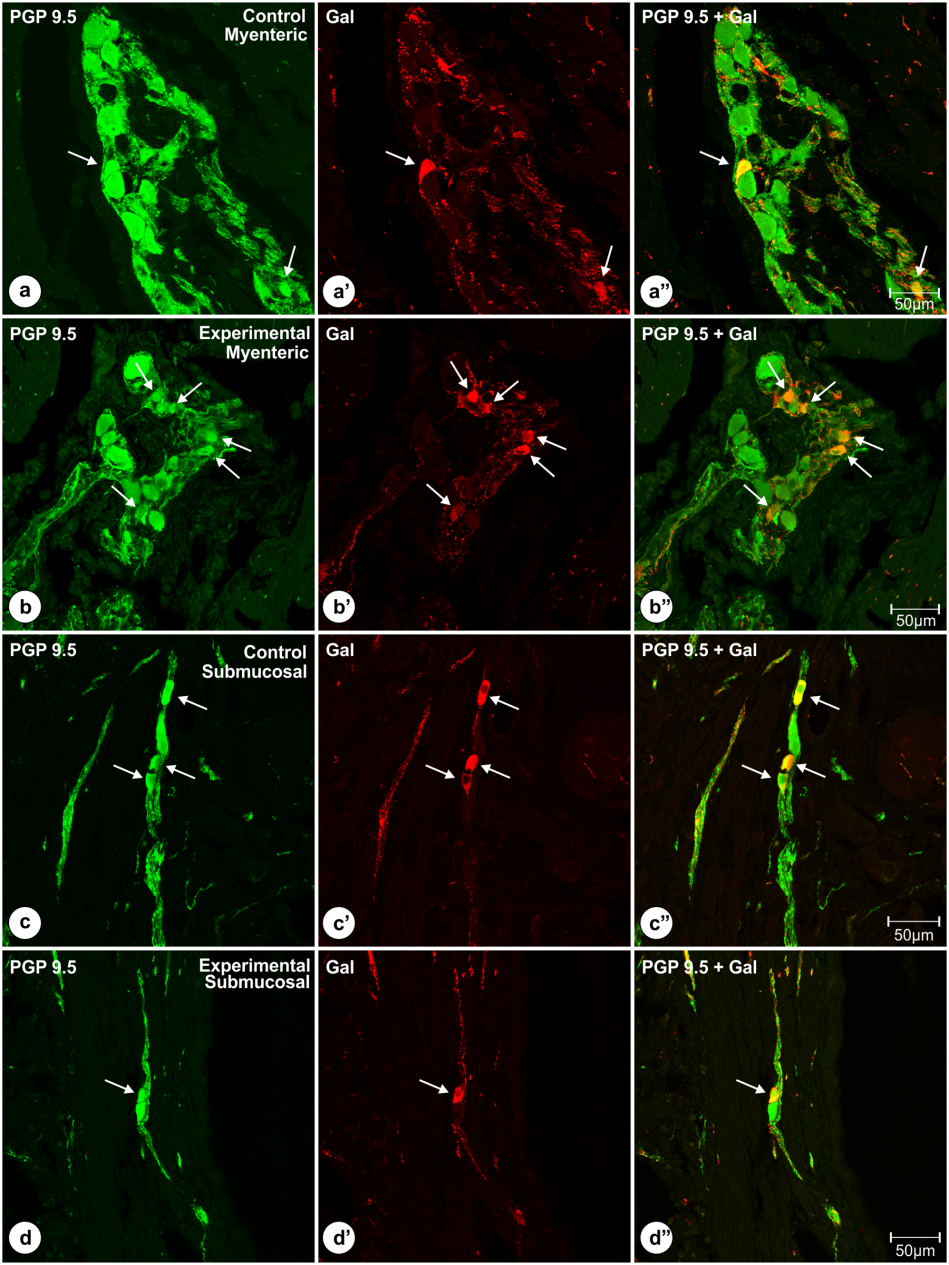
Double immunolabeled (PGP 9.5 and Gal) perikarya of the pyloric orifice wall. Set of microphotographs showing sections of the pyloric orifice wall taken from the control and experimental pigs of the subgroup H and double-immunolabeled with antibodies against PGP 9.5 (a, b, c, d) and galanin (a’, b’, c’, d’). Some of the myenteric plexus perikarya (arrows) of the control (a, a’, a”) and experimental (b, b’, b”) animals simultaneously co-expressed immunoreactivity to PGP 9.5 (a, b) and galanin (a’, b’). The number of PGP 9.5+/Gal+ neurons was higher in the experimental animals and the difference was statistically significant. Some of the submucosal neurons (arrows) of the control (c, c’, c”) and experimental (d, d’, d”) animals simultaneously co-expressed immunoreactivity to PGP 9.5 (c, d) and galanin (c’, d’), and these percentages did not differ significantly between both groups of animals. Pictures (a”, b”, c”, d”) show the overlap of both fluorescence channels (PGP 9.5—green, Gal—red). Scale bars are included in the pictures.

**Fig 5:**
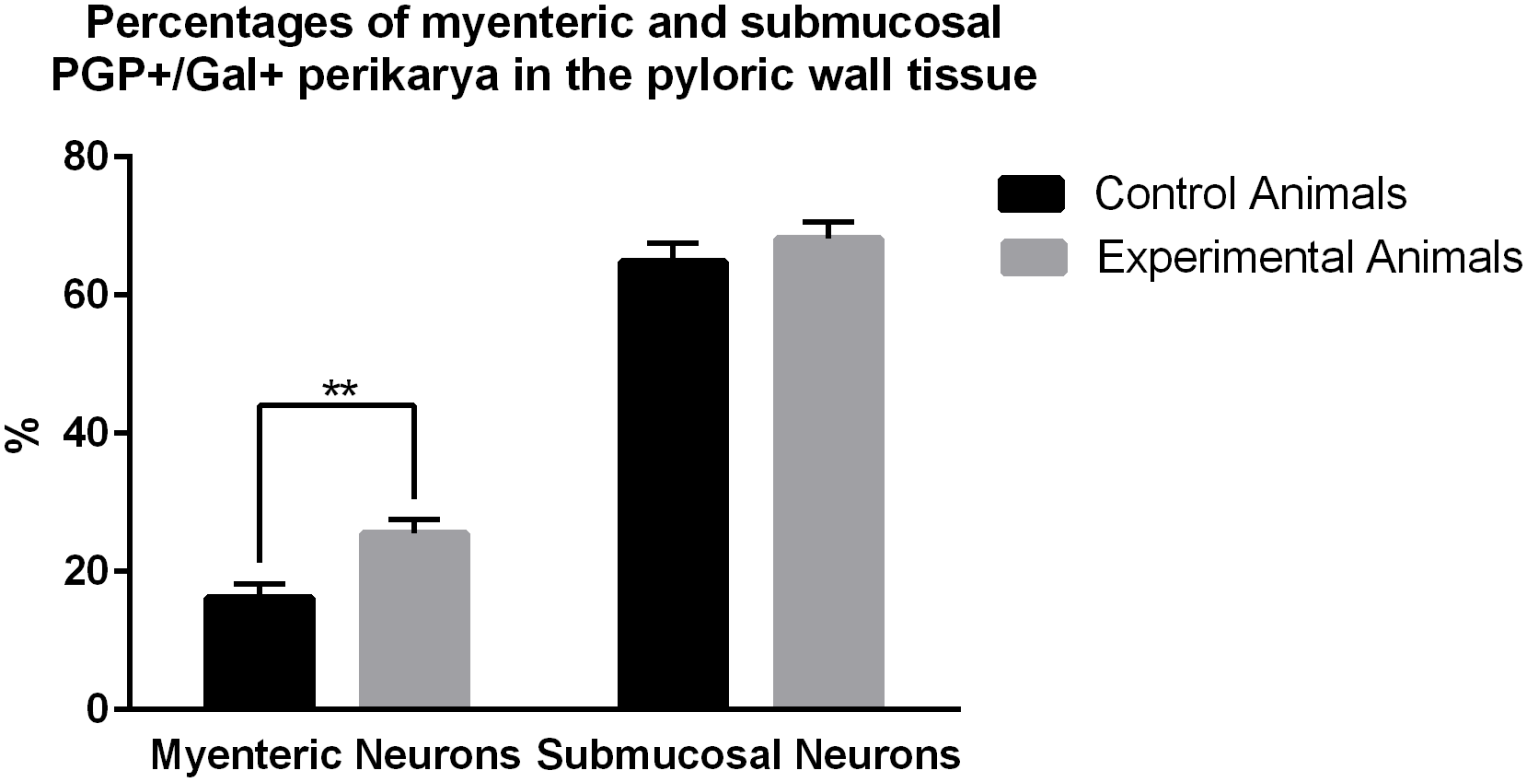
Percentages of myenteric and submucosal PGP+/Gal+ perikarya in the pyloric wall tissue. Graph showing percentages of the myenteric and submucosal PGP+/Gal+ perikarya in the pyloric wall samples collected from the control and experimental animals of the subgroup H. Statistically significant differences between the control and experimental animals are marked by asterisks, ** P ≤ 0.005.

**Fig 6:**
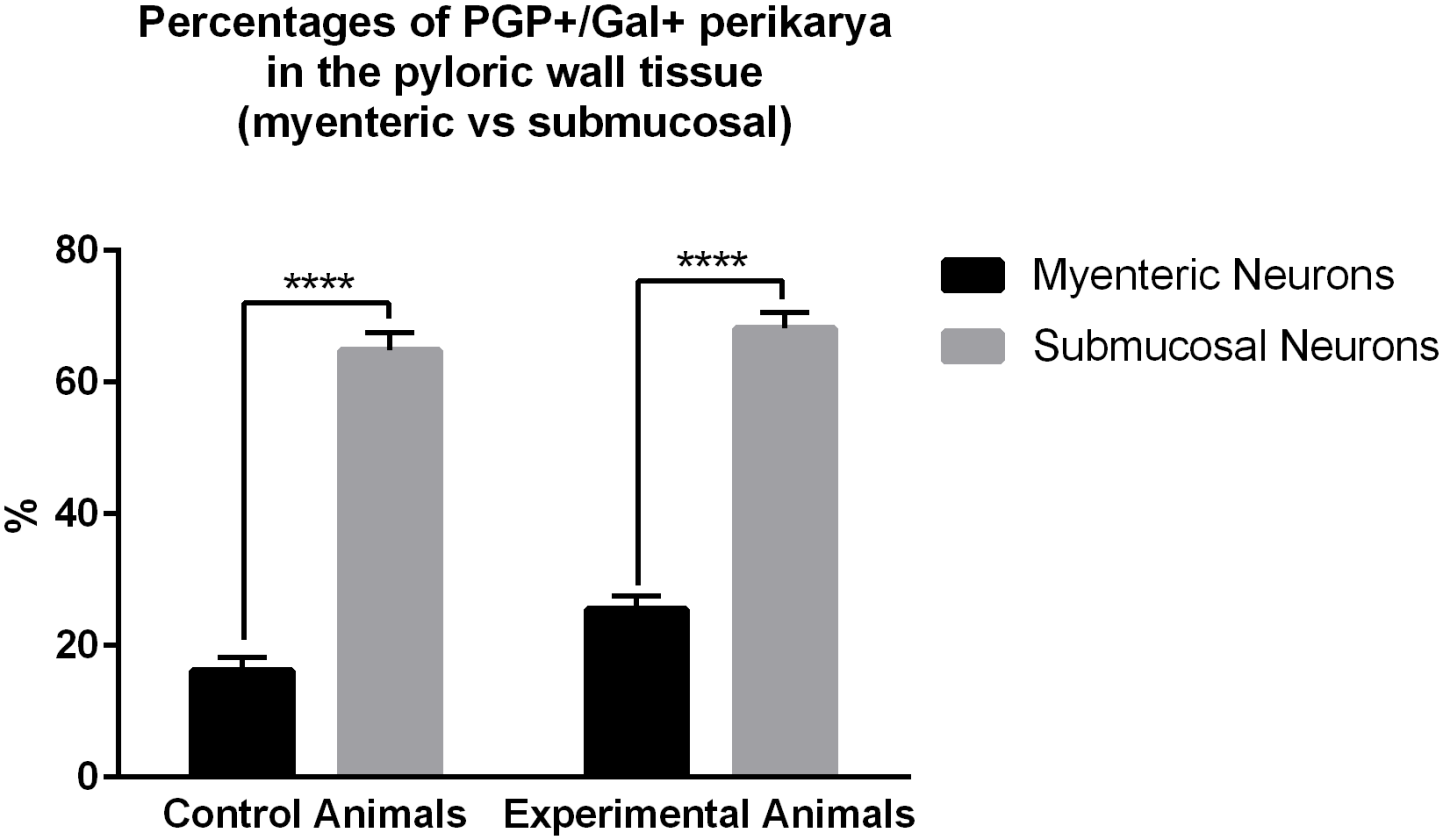
Differences between the percentages of myenteric and submucosal PGP+/Gal+ perikarya in the pyloric wall tissue. Graph presenting differences between the percentages of myenteric and submucosal PGP+/Gal+ perikarya in the control and experimental pigs of the subgroup H. The number of galaninergic submucosal neurons was significantly higher in both groups of animals. Statistically significant differences between numbers of myenteric and submucosal neurons are marked by asterisks, **** P ≤ 0.0001.

Quantitative analyses revealed that in the control animals 16.14 ± 2.06% of myenteric plexus neurons (Fig 4a, 4a’ and 4a”) exhibited immunoreactivity to Gal (Fig 4a’), while in experimental pigs (Fig 4b, 4b’ and 4b”) this number amounted to 25.5 ± 2.07%, and the difference was statistically significant (Fig 5). The number of the pyloric wall submucosal Gal-immunoreactive perikarya was 64.84 ± 2.74% in the control (Fig 4c, 4c’ and 4c”) and 68.16 ± 2.49% in experimental (Fig 4d, 4d’ and 4d”) animals, and this difference was not statistically significant (Fig 5). Statistical comparison of the number of PGP 9.5/Gal-positive submucosal and myenteric perikarya revealed significantly greater number of submucosal galaninergic neurons in both groups of animals (Fig 6).

**Fig 7:**
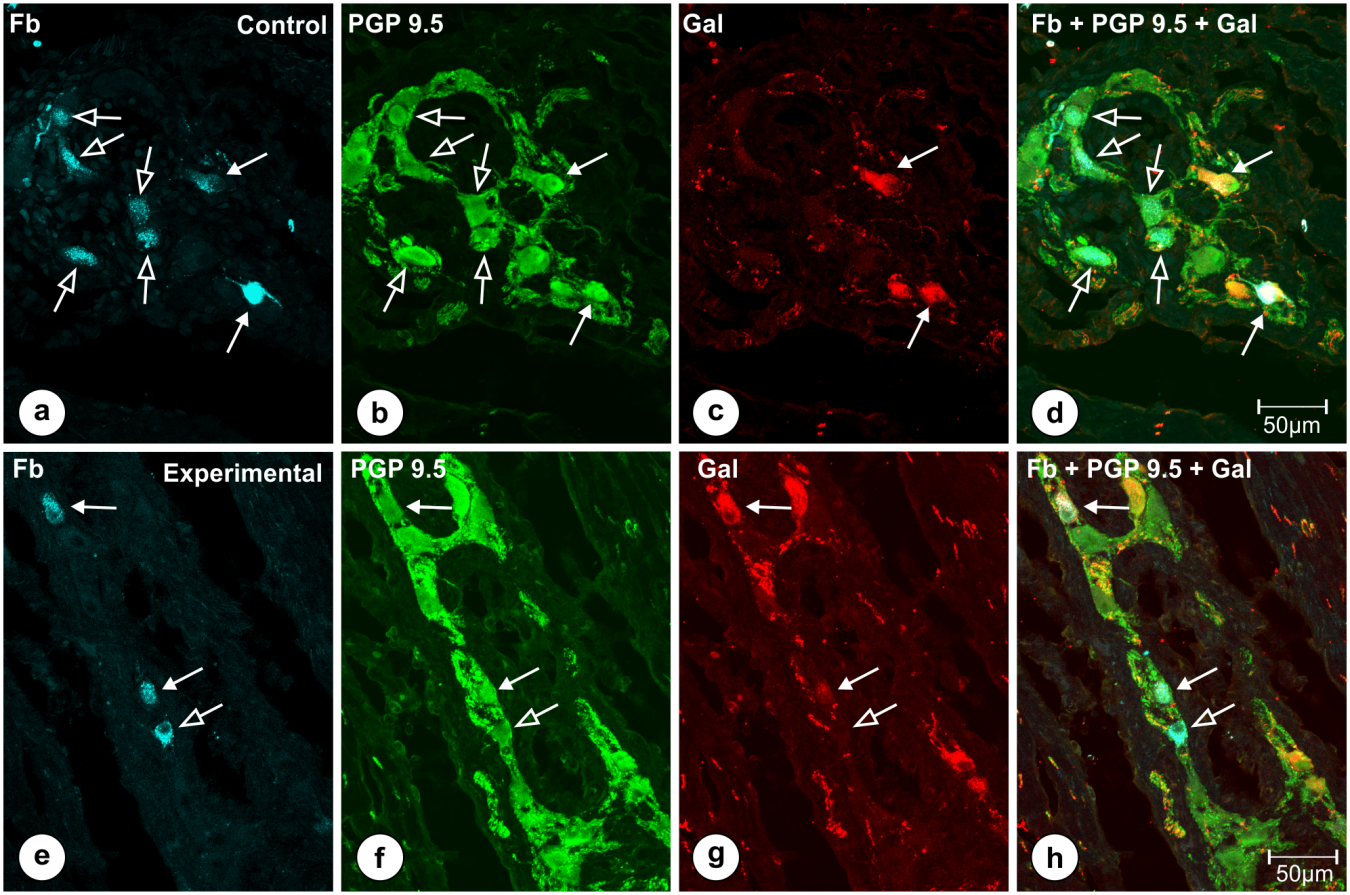
FB-positive neurons of the stomach antrum double-immunostained for PGP 9.5 and Gal. Set of microphotographs showing stomach antrum sections with FB-positive neurons (a, e) from the animals of subgroup T and double-immunostained with antibodies against PGP 9.5 (b, f) and galanin (c, g). Some of FB-positive perikarya (solid arrows) in the control (a) and experimental (e) animals simultaneously co-expressed immunoreactivity to PGP 9.5 (b, f) and galanin (c, g), while the other traced neuronal somata (empty arrows) were devoid of galanin immunoreactivity. Differences in the number of FB+/PGP 9.5+/Gal+ neurons did not differ significantly between both groups of the animals. Pictures (d, h) show the overlap of all three fluorescence channels (FB—blue, PGP 9.5—green, Gal—red). Scale bars are included in the pictures.

**Fig 8:**
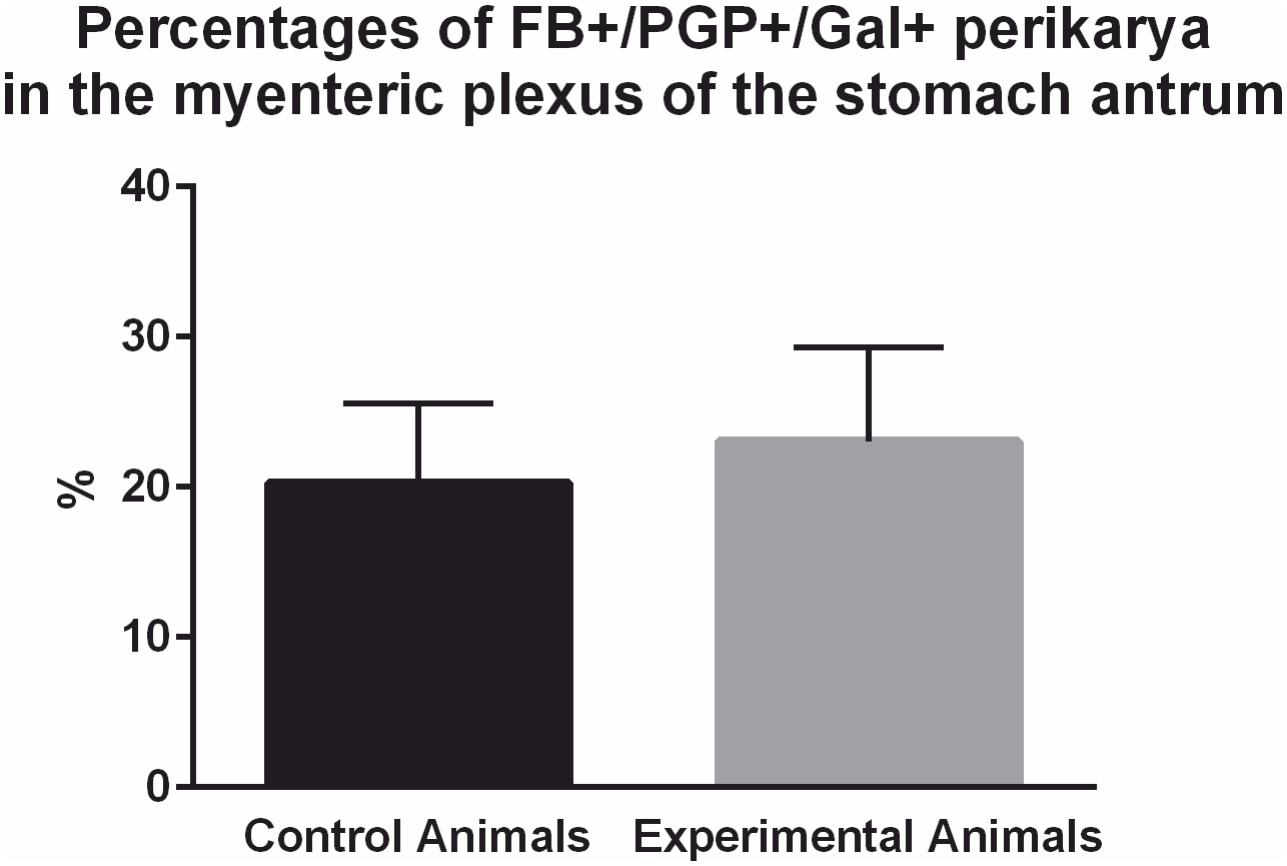
Percentages of FB+/PGP+/Gal+ perikarya in the myenteric plexus of the stomach antrum. Graph presenting percentages of FB-positive neurons supplying the pyloric sphincter in the control and experimental animals of the subgroup T which simultaneously co-expressed immunoreactivity to PGP 9.5 and Gal. The differences between the control and experimental animals were statistically insignificant.

The analysis of the gastric antrum sections (Fig 1—blue frame) taken from the pigs of subgroup T revealed that the traced cells supplying the pylorus were localized exclusively in the myenteric plexus. Double immonolabeling with the mixture of anti-PGP 9.5/Gal antibodies revealed that in the control animals 20.3 ± 2.163% of Fast Blue positive cells simultaneously co-expressed immunoreactivity to PGP 9.5 and Gal (Fig 7a, 7b, 7c and 7d), while in the experimental pigs (Fig 7e, 7f, 7g and 7h) this number amounted to 23.06 ± 6.232% and the difference was not statistically significant (Fig 8).

The analysis of the control staining sections did not reveal any positive signal in none of the control tissue slide what confirmed the specificity of the staining procedure.

Because of some information on the erroneous and uncertain reaction of antibodies against galanin receptors [38] and due to the specificity of the species studied (there are no specific antibodies designed to porcine receptors), the authors decided to verify changes in the galanin receptors expression (in the pyloric wall tissue of the subgroup M animals) only by using the precise and reliable Real-Time PCR technique.

**Fig 9:**
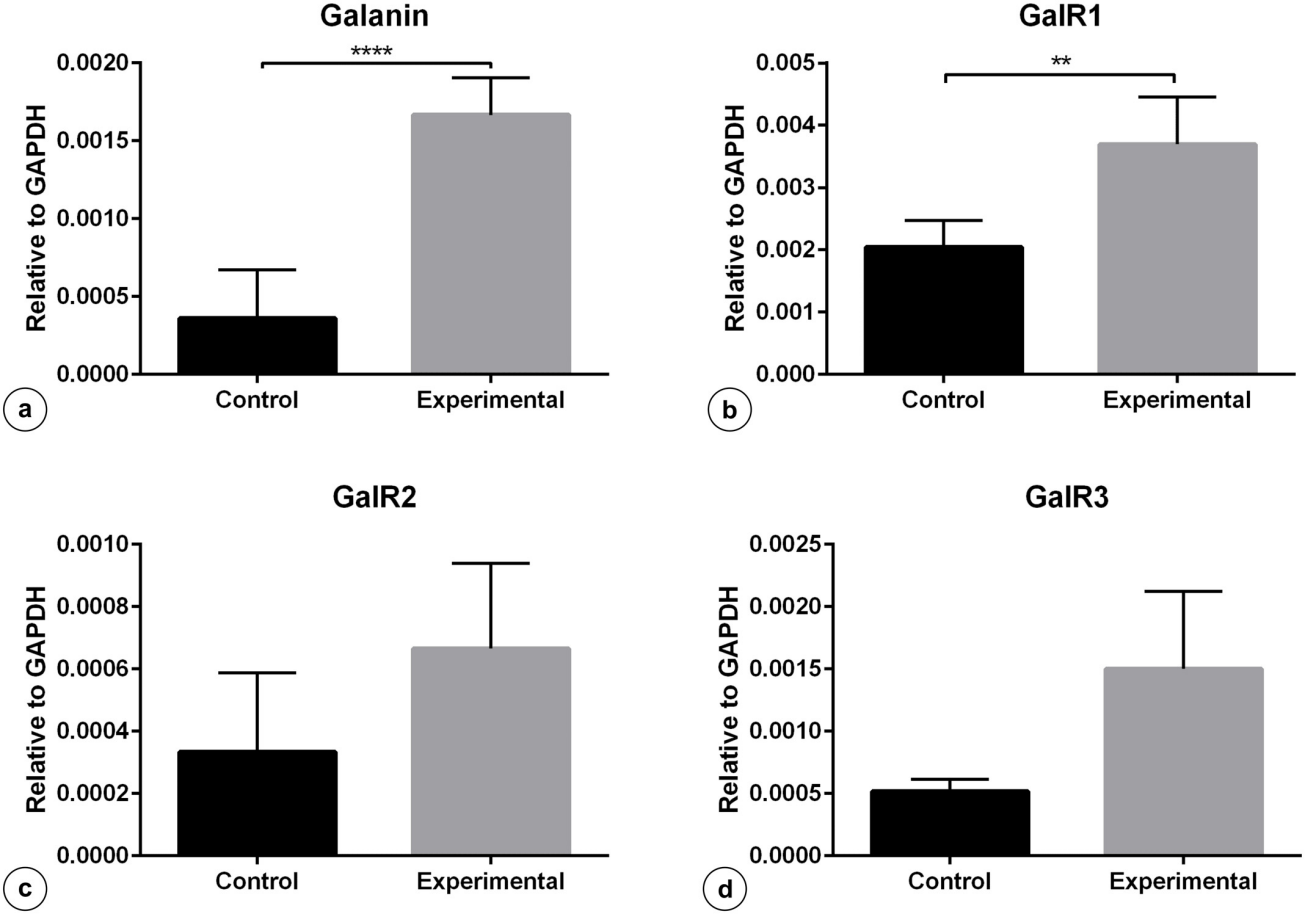
Expression of mRNA encoding Gal, GalR1, GalR2, GalR3 in the pyloric wall tissue. Expression of Gal (a), GalR1 (b), GalR2 (c) and GalR3 (d) mRNA in the pyloric wall tissue collected from the control and experimental animals of subgroup M. Levels of Gal, GalR1, GalR2, GalR3 mRNA were measured by Real-Time PCR. The data obtained from each sample were normalized to GAPDH. Relative quantities (RQ) of mRNA were analysed using the comparative Ct method. Each cDNA sample was amplified in triplicate and all data are expressed as the mean ± S.E.M., **** P ≤ 0.0001; ** P ≤ 0.005 (vs the control animals).

The results of quantitative Real-Time PCR revealed statistically significant increase in the expression of mRNA encoding galanin (Fig 9a) and GalR1 receptor (Fig 9b) in the pyloric wall tissues (Fig 1—violet circles and frames) of the experimental animals in subgroup M, while the expression of GalR2 (Fig 9c) and GalR3 (Fig 9d) receptors was not changed in a statistically significant way.

## Discussion

The present results have revealed for the first time that gastric antral ulcerations are associated with plastic changes dealing with the expression of galanin in gastric enteric neurons and galanin receptors in the gastric tissue in a mammalian species, the pig. In the animals with antral ulcerations the number of galanin-immunoreactive myenteric neurons localized within the pyloric wall was increased in relation to that found in the control pigs, while the number of Gal-positive myenteric gastric descending neurons supplying the pyloric sphincter remained unchanged. Most of the myenteric plexus neurons were localized deep in muscular layers of the porcine pyloric sphincter wall, what is in agreement with earlier data reporting similar distribution of myenteric neurons in the pyloric orifice wall [15]. Interestingly, there were also no statistically significant differences in the number of pyloric wall Gal-positive submucosal neurons between both groups of the animals. The specific pattern of galanin immunoreactivity observed in the neurons seems to correlate with the cytoplasmic vesicles and Golgi apparatus subcellular localization of the studied protein [39]. Real-Time PCR technique revealed that in the pyloric wall tissue of the experimental pigs mRNAs encoding galanin and GalR1 receptor were up-regulated, while differences in the expression of GalR2 and GalR3 receptors were not statistically significant.

Peptides are crucial for the regulation of inflammatory processes [40]. Galanin as well as its receptors, especially GalR1, are undoubtedly key players in the cross-talk between the neuroendocrine and immune systems [23]. Changes in the expression of galanin and its receptors have been observed in neurons of the central as well as the peripheral nervous system in many inflammatory experiments [1]. Although the great majority of studies have indicated the upregulation of galanin in peripheral tissues covered by the inflammatory processes [41–46], including the tissues of the porcine gastrointestinal tract [47–51], there are also some contributions reporting its downregulation [44;52]. The increased number of galanin-positive neurons in the myenteric plexus of the pyloric wall observed in the present experiment clearly indicates the influence of antral ulcers on these distanced pyloric perikarya. The authors assume that the weaker immunofluorescence observed in occasional myenteric perikarya in the experimental animals might result from the fact that some larger neurons had already started galanin production and thus cellular levels of the peptide were low. It should be emphasized that publications cited above reported the upregulation of galanin in the nerve structures found in the inflamed gastrointestinal tract tissues, while the present results indicate the upregulation of galanin in the neurons localized in tissues which were not directly covered by the inflammatory process. The upregulation in the galanin mRNA expression in the pyloric wall tissue of the experimental animals revealed by Real-Time PCR technique is indicative of the reaction of cells in the distant pyloric tissue to the antral ulcers. The mechanism of activation of the pyloric myenteric neurons (to induce galanin upregulation) by antral ulcerations should be elucidated, however, based on the available information the following possibilities could be hypothesized. First, the activation of the myenteric neurons could be accomplished by cytokines released from immune cells that follow to the ulcer’s inflammatory site [53]. The ENS neurons are known to express receptors for pro-inflammatory cytokines which can affect these cells [54] thus resulting in galanin upregulation. The other possibility of myenteric cells activation could be the involvement of extrinsic nerve reflexes, which are known to participate in the intestinal inflammatory interactions [55]. The stomach [16;56] including the pylorus [15;36;57;58] are known to be widely innervated by extrinsic nerves which originate exactly in the nodose ganglia, dorsal motor nuclei of the vagus nerve as well as in the coeliac superior mesenteric ganglion. Since the experimental vagal deafferentiation was followed by a significant delay in the gastric emptying in pigs [59] and vagal activation promoted the transpyloric flow by reducing the pyloric resistance [60;61], the involvement of extrinsic vagal neural reflexes in the pyloric wall myenteric neurons activation seems to be quite possible. Moreover, the extrinsic innervation was submitted to control the release of galanin from the ileum [62] and adrenal glands [63] in pigs, what can be directly linked with upregulation of Gal in myenteric neurons observed in our experiment. The finding that the expression of galanin was unchanged in the gastric descending neurons supplying the pyloric sphincter further supports the idea of extrinsic reflexes participation in the precise activation of myenteric pyloric neurons to induce alterations in their chemical phenotype. It seems to be reasonable to assume, that pro-inflammatory cytokines released by immune cells infiltrating the ulcer would also activate the gastric descending myenteric neurons, which incidentally, are localized closer to the pathologically changed area (in the stomach antrum). Gastric descending neurons are known to have inhibitory function to the pyloric sphincter [64]. Although, the number and distribution of gastric descending nerve cells supplying the pyloric sphincter were significantly changed in pigs with antral ulcerations [25], the present results seem to exclude the direct participation of galanin in these descending nerves plasticity. Finally, the possibility that some of the galanin-positive myenteric neurons localized within the pylorus are ascending nerve cells projecting to the antrum and they are activated directly by the ulcer wound cannot be excluded. However, physiological experiments clearly demonstrated that direct focal electrical stimulation of myenteric neurons and muscles in the pylorus induced considerably greater responses of sphincteric muscles than those evoked by the stimulation of ascending and descending neurons in the gastric antrum [64]. This observation clearly suggests the overriding role of myenteric neurons localized within the exact pylorus in the regulation of the sphincteric muscle activity and contributes to the idea that the vast majority of Gal-positive neurons found in the myenteric plexus of the pyloric wall supply the sphincteric musculature. Galanin released from peripheral nerve endings of these activated Gal-positive myenteric neurons can directly influence smooth muscles of the pyloric sphincter. Data on the direct response of gastrointestinal smooth muscles to galanin are vague, which seems to be associated with the species studied and the part of the gastrointestinal tract explored. Although galanin has been found to relax [65–68] or contract [8;9;65;69–74] gastrointestinal smooth muscles, or even inhibit circular and excite longitudinal smooth muscle cells (as found in the porcine ileum [75]), its direct interactions with smooth muscles of the gastrointestinal tract are still uncertain. More recent studies have reported the presence of GalR2 receptor (which is the only excitatory galanin receptor subtype) in gastrointestinal smooth muscles cells, and suggested the contractile effect of its activation by galanin [76;77]. In view of gastric emptying problems occurring in patients with antral ulcers and considering the present results, further physiological studies on the sphincteric muscles response to galanin seem to be of particular interest.

Despite the fact that the population of submucosal pyloric neurons was numerous in both groups of animals, interestingly, they did not respond with the increased expression of galanin to antral ulcerations.

Earlier studies performed in different species [43;45;46], including the pig [47;48;50;51;78;79] have demonstrated an increased number of myenteric and submucosal galanin positive neurons in animals with gastrointestinal inflammation, what might result from the direct reaction of the mucosa (and its innervation) to pathological processes. Our discrepant findings could be explained by the type of the tissue damage induced in the present experiment and the remoteness of the pyloric tissue from the site of injury. The ulcer tissue damage evoked in the present study was restricted to the surface area of 1–2.1 cm in diameter and deeply penetrated into the muscular layers, without affecting the large areas of the mucosa, as found in other studies, in which inflammatory processes were induced chemically [45] or by infection with enteric pathogenic bacteria [48;50;51;78;79]. What is more, the deeply penetrating ulcers extensively damaged the muscular layer. The destruction of muscular tissues of the gastrointestinal tract strongly influenced the activity of myenteric neurons [54], what can be linked to the upregulation of Gal exclusively in the myenteric neurons in our experiment.

The results of the Real-Time PCR technique with primers designed to porcine GalR1, GalR2 and GalR3 receptor subtypes revealed a statistically significant increase in the expression of only GalR1 in the pyloric wall tissue of the experimental pigs in relation to that found in the control animals, while the differences in the expression of GalR2 and GalR3 were not statistically significant between both groups.

Considering the gastrointestinal tract, GalR1 is mainly expressed by extrinsic [31;80] and intramural [6;30;32;81–84] neurons as well as by enterochromaffin-like and epithelial cells [84;85]. The GalR1 is suggested to facilitate galanin actions on gastrointestinal functions (such as motility and secretion) mainly by modulation of other neurotransmitters release [86]. Its expression has been frequently observed in myenteric cholinergic neurons (immunoreactive to acetylcholine transferase and vesicular acetylcholine transporter), which are known to be the intramural excitatory neurons [30;32;84]. This receptor has been proposed to mediate many inhibitory actions of galanin [80;87]. GalR1 pathways are strongly involved in the gastric [31] and jejunal [32] motility regulation. The expression of GalR1has been found to be upregulated in peripheral tissues in almost all experimental inflammatory models [1], what is in agreement with results obtained in our study. However, some differences seem to be worth of notice. Firstly, most of the gastrointestinal experiments described by Lang and Kofler (2011) were related to the intestines, but not to the stomach. Secondly, all these inflammatory processes were induced by infections with enteric pathogens or application of chemical compounds, and such procedure certainly affected the wide areas of the mucosa. On the other hand, the upregulation of GalR1 was found to be confined to the epithelial cells of the affected mucosa [85;88–91]. In our experiment, the pyloric mucosa was not directly affected by the acute inflammatory process what suggests a minor participation of epithelial cells in the receptor upregulation.

Interestingly, in the previous study we have observed a significant decrease in the expression of galanin, GalR1, GalR2 and GalR3 receptors (in the mucosa, tunica muscularis and lymphocytes) in the porcine descending colon wall affected by dysentery associated colitis [37]. Although both of our experiments were conducted in pigs, the colonic tissues examined in the previous study were directly affected by severe inflammation and deep destruction of the colonic wall tissue was observed. Thus, some structures expressing the peptide and its receptors could had been destroyed, what might be the reason for such divergent results obtained in our both contributions. Furthermore, the divergent reaction of porcine intestinal circular and longitudinal muscles to galanin [75] indicates the complexity of the galanin associated actions in swine gastrointestinal tract under physiological and pathological conditions. All these facts suggest the need for further studies on GalR1 function in the gastrointestinal tract in the pig, a highly valuated animal.

Galanin receptor 2 is the only excitatory receptor, the activation of which results in direct gastrointestinal smooth muscle contractions [76;77]. Its expression was determined in each part of the gastrointestinal tract, however, the highest levels of the mRNA were detected in the stomach [6]. Besides the smooth muscle cells [76;77], the GalR2 was also found to be present in myenteric neurons of the canine and sheep stomach [83], and in some sensory neurons [92–94]. Peripheral GalR2 is additionally considered to play a role in modulation of pain [95;96] and vagal afferent mechanosensivity [80]. Although we have not observed statistically significant changes in the expression of GalR2 in the pyloric wall tissue of the experimental pigs, its participation in the regulation of the pyloric activity during ulcer disease cannot be excluded. Since GalR2 is the only excitatory galanin receptor subtype and the only receptor mediating direct smooth muscles contractions in the gastrointestinal tract [76;77], its activation by increased amounts of galanin in the pylorus of the experimental animals could be directly related with the gastric emptying problems in patients with antral ulcerations, but the role of this phenomena needs to be explained in further investigations.

Galanin receptor 3, similarly as Gal R1, mediates inhibition of adenyl cyclase activity and activates an inward of K+ current [97]. Although data concerning the distribution and function of GalR3 are sparse, there are some publications describing its expression in the gastrointestinal tract [6;97;98] including myenteric neurons of the stomach [83]. It should be noted, that most of these reports have underlined the low or extremely low concentrations of GalR3 mRNA in the gastrointestinal tissues. Studies on the involvement of GalR3 in inflammatory processes are occasional and only its role in the regulation of microvasculature and oedema formation in dermatitis has been described [99]. The present study has not revealed statistically significant changes in GalR3 expression in the pyloric wall tissues of the experimental pigs. The lack of data on the involvement of GalR3 in the smooth muscle activity and inflammatory processes in the gastrointestinal tract suggests its minor role in the pyloric wall response to antral ulcers.

It can be assumed that the changes in the galanin and GalR1 receptor expressions in the pyloric wall tissue observed in the animals with antral ulcerations could have different ethology and significance from those described in other gastrointestinal inflammatory experiments. The increased expression of mRNA encoding galanin and GalR1 as well as the increased number of the myenteric neurons immunoreactive to Gal in the pyloric wall tissue distant from the ulcer injury, undoubtedly demonstrate the participation of galanin in the plasticity of sphincteric nerve regulation in subjects with antral ulcerations. The neuronal “galaninergic” regulatory process seems to be highly specialized, which is supported by the lack of changes neither in the number of Gal-positive submucosal perikarya localized within the pylorus nor in the gastric descending neurons supplying the pyloric sphincter. Furthermore, the GalR2 and GalR3 receptors seem to be not directly involved in such “galaninergic” tissue regulation. The present results provide new insights into the participation of galanin in the neural regulation of the pylorus function in mammals with ulcerative disease. The relationship between galanin and GalR1 upregulation, and the function of the pyloric sphincter should be further studied in terms of gastric emptying problems in patients with antral ulcerations.
